# Triterpenoids from *Ainsliaea latifolia* and Their Cyclooxyenase-2 (COX-2) Inhibitory Activities

**DOI:** 10.1007/s13659-019-00228-x

**Published:** 2019-11-30

**Authors:** Wen-Lin Yuan, Xue-Yun Dong, Zheng-Rui Huang, Si-Jia Xiao, Ji Ye, Xin-Hui Tian, Hui-Liang Li, Yun-Heng Shen, Wei-Dong Zhang

**Affiliations:** 1 Department of Phytochemistry, School of Pharmacy, Naval Medical University (Second Military Medical University), Shanghai, 200433 China; 2 Interdisciplinary Science Research Institute, Shanghai University of Traditional Chinese Medicine, Shanghai, 201203 China; 3 Department of Applied Chemistry, Xi’an University of Technology, Xi’an, 710048 China; 4 School of Pharmacy, Fujian University of Traditional Chinese Medicine, Fujian, 350108 China

**Keywords:** *Ainsliaea latifolia*, Triterpenoids, COX-2, Cucurbitane

## Abstract

**Abstract:**

Eight new triterpenoids were isolated from *Ainsliaea latifolia.* The structures of these compounds were elucidated by interpretation of spectroscopic data, including HRESIMS and NMR data. Compounds **4**–**6** are identified as rare trinorcucurbitane or tetranorcucurbitane triterpenoids. The absolute configurations of compounds **1** and **2** were confirmed by Snatzke’s method. All compounds were evaluated for their inhibition against cyclooxyenase-2 (COX-2), in which compound **4** showed significant inhibitory effect against COX-2 with IC_50_ value of 3.98 ± 0.32 μM, comparable to that of positive control NS-398 (IC_50_ 4.14 ± 0.28 μM).

**Graphic Abstract:**

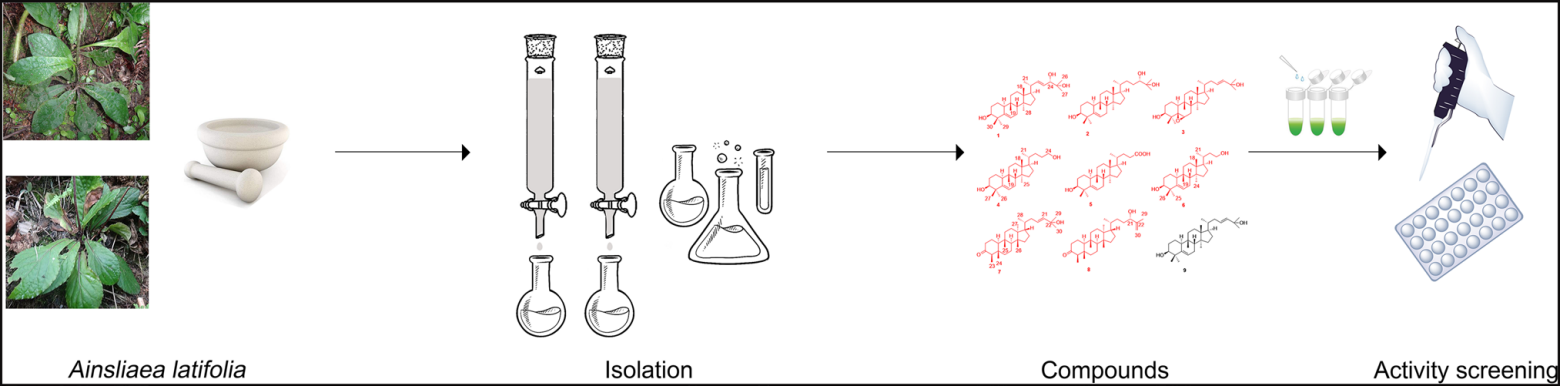

**Electronic supplementary material:**

The online version of this article (10.1007/s13659-019-00228-x) contains supplementary material, which is available to authorized users.

## Introduction

The genus *Ainsliaea* (Compositae), a medicinally important genus in traditional Chinese medicine, comprises about 70 species worldwide, in which most *Ainsliaea* plants are distributed in Southeast Asia. Previous investigations have reported the presence of sesquiterpenoids, sesquiterpene lactone dimers, triterpenoids, steroids and flavonoids in *Ainsliaea* species [1–3]. Some of them exhibited diverse biological activities, including cytotoxic, antiviral, antibacterial and anti-inflammatory activities [4–6].

*Ainsliaea latifolia* grows mainly in the southwest of China and has long been used as a folk medicine for the treatment of rhumatism, traumatic injuries, edema, stomachache, and anorexia [7]. In *Ainsliaea* species, sesquiterpenoids are usually considered as characteristic chemical constituents. However, in our study of the chemical constituents from *A. latifolia*, eight new triterpenoids **(1–8**) and one known triterpenoid (**9**) were isolated and identified from the whole plants of *A. latifolia.* Herein, we described the isolation and structural elucidation of compounds **1**–**8**, as well as their inhibition against cyclooxyenase-2 (COX-2).

## Results and Discussion

**Fig. 1:**
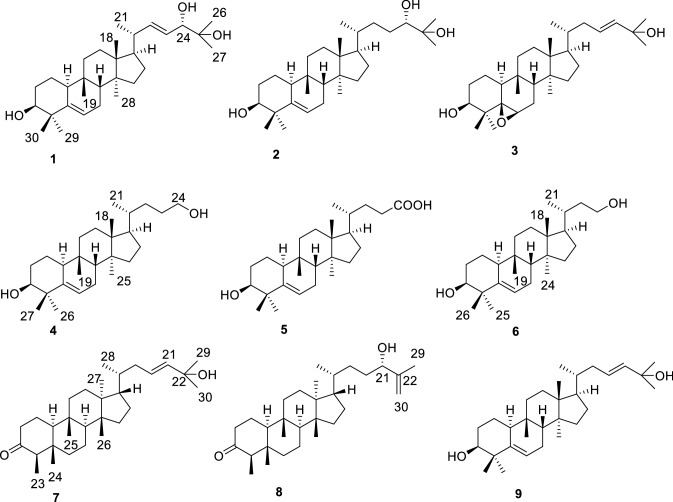
Chemical structures of **1–9**

The CHCl_3_-soluble of the EtOH-H_2_O (80:20, v/v) extract of *A. latifolia* was purified by repeated column chromatography (CC) over silica gel, Sephadex LH-20, and semi-preparative HPLC to yield eight new and one known compounds. By comparison of their NMR and MS data with the published references, the known compound **9** was then identified as one triterpenoid cucurbita-5,23-diene-3*β*,25-diol (**9**) [8]. The structures of eight new triterpenoids were determined by analysis of HRESIMS and NMR spectroscopic data (Fig. 1).

**Fig. 2:**
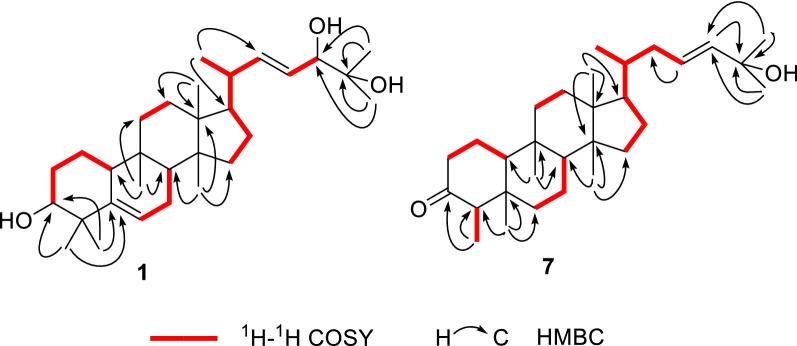
Selected ^1^H–^1^H COSY and HMBC correlations of **1** and **7**

**Fig. 3:**
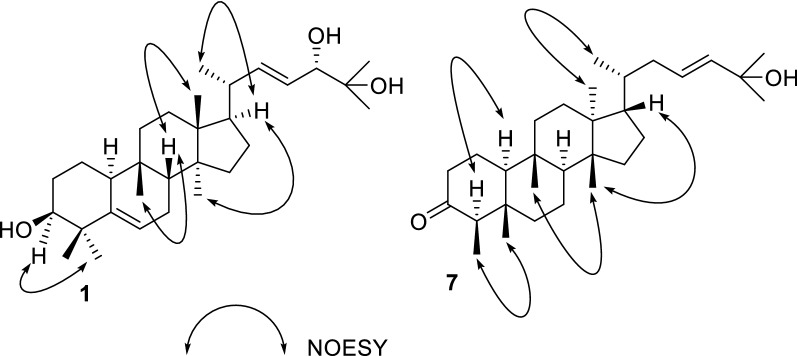
Key NOESY correlations of **1** and **7**

**Fig. 4:**
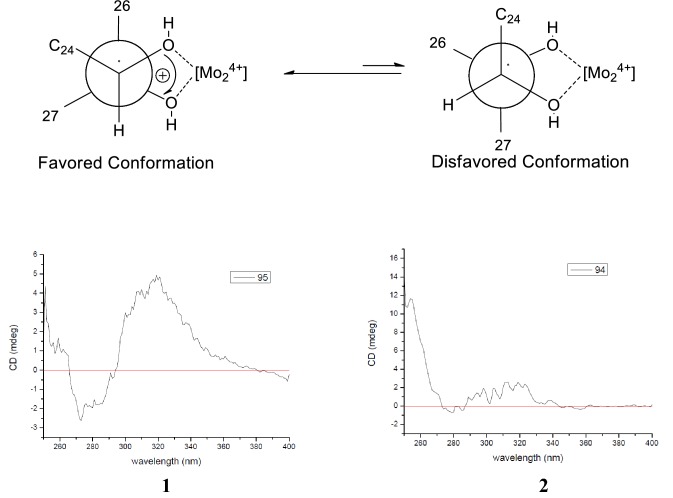
Conformations and ICD spectra of the Mo_2_^4+^ complex of **1** and **2** in DMSO

Compound **1** was isolated as white solid. Its molecular formula (C_30_H_50_O_3_), ascertained via high resolution ESI–MS analysis, indicated six degrees of unsaturation. The ^1^H NMR spectrum of **1** (Table 1) exhibited signals for three olefinic protons at *δ*_H_ 5.59 (2H), 5.42 (1H, m), two oxygenated methine groups at *δ*_H_ 3.83 (1H, d, *J* = 7.1 Hz), 3.47 (1H, brt, *J *= 2.5 Hz), eight methyl groups (*δ*_H_ 1.20, 1.14, 1.13, 1.02, 1.00, 0.92, 0.87, 0.81). The ^13^C NMR spectrum revealed the presence of thirty carbon signals including four olefinic carbons at *δ*_C_ 141.2, 141.3, 125.7 and 121.4, three oxygenated carbons at *δ*_C_ 79.7, 76.6 and 72.9, and eight methyl carbons at *δ*_C_ 28.0, 27.2, 26.3, 25.4, 23.7, 20.4, 17.8 and 15.7. The other carbon signals were assigned to seven methylenes, four methines, and four quaternary carbons. A comparison of these carbon resonances with those of the related cucurbitane-type triterpenoids suggested that **1** possessed the same cucurbitane skeleton, and the differences between the spectroscopic data of **1** and those of known compound **9** were primarily the observation of an oxymethine and the absence of a methylene. In the ^1^H–^1^H COSY spectrum of **1**, two mutual coupling olefinic protons exhibited the correlations with H-20 and the oxygenated methine proton at *δ*_H_ 3.83 (Fig. 2), respectively, ascribing a double bond to C-22 and C-23 positions. The HMBC correlation (Fig. 2) of CH_3_-21 with the olefinic carbon at *δ*_C_ 141.3 confirmed the above deduction. Also, the observation of HMBC correlations from CH_3_-26 and CH_3_-27 to C-24 (*δ*_C_ 79.7) and the oxygenated quaternary carbon at *δ*_C_ 72.9 supported the hydroxyl substituents at C-24 and C-25 positions. The absolute configuration of C-24 in **1** was assigned using the Snatzke’s method [9, 10]. Metal complex of compound **1** in DMSO gave a significant induced CD spectrum (ICD) (Fig. 4), in which the positive cotton effect observed at 315 nm permitted the assignment of a 24*S* configuration for **1**. The relative configurations of other stereocenters of **1** were established to be identical to those of known compound **9** due to NOESY experiment (Fig. 3). Thus, the structure of compound **1** was identified as cucurbita-5, 22-diene-3*β*, 24*S*, 25-triol.

**Table 1:** ^1^H (500 MHz) and ^13^C (125 MHz) NMR spectroscopic data of compounds **1**–**4** in CDCl_3_

No.	Compound **1**		Compound **2**		Compound **3**		Compound **4**	
	*δ*_C_	*δ*_H_ (*J* in Hz)	*δ*_C_	*δ*_H_ (*J* in Hz)	*δ*_C_	*δ*_H_ (*J* in Hz)	*δ*_C_	*δ*_H_ (*J* in Hz)
1	21.1	1.58, m 1.47, m	21.1	1.58, m 1.47, m	19.9	1.76, m 1.63, m	21.1	1.57, m 1.46, m
2	28.9	1.69, m 1.46, m	28.9	1.69, m 1.46, m	27.5	1.87, m 1.12, m	28.9	1.69, m 1.46, m
3	76.6	3.47, brt (2.5)	76.6	3.47, brt (2.5)	78.5	3.47, s	76.6	3.47, s
4	41.4	–	41.4	–	39.4	–	41.4	–
5	141.2	–	141.2	–	66.8	–	141.2	–
6	121.4	5.59, overlap	121.5	5.59, d (5.9)	53.2	3.16, d (5.8)	121.5	5.59, d (5.7)
7	24.3	2.39, m 1.79, m	24.3	2.39, m 1.79, m	22.7	2.21, m 1.71, m	24.4	2.39, m 1.79, m
8	43.6	1.76, m	43.6	1.76, m	42.4	1.67, m	43.6	1.76, m
9	34.5	–	34.4	–	33.9	–	34.5	–
10	37.8	2.26, d (12.1)	37.8	2.26, d (12.3)	35.2	2.21, m	37.8	2.26, d (12.5)
11	32.3	1.66, m 1.43, m	32.3	1.64, m 1.43, m	33.6	1.63, m 1.32, m	32.3	1.66, m 1.43, m
12	30.4	1.71, m	30.4	1.67, m	30.1	1.64, m	30.4	1.65, m
		1.46, m		1.46, m		1.46, m		1.46, m
13	46.3	–	46.2	–	45.8	–	46.2	–
14	49.2	–	49.1	–	49.1	–	49.2	–
15	34.8	1.20, m 1.15, m	34.7	1.20, m 1.14, m	34.6	1.23, m 1.13, m	34.7	1.20, m 1.14, m
16	28.2	1.24, m 1.16, m	27.9	1.24, m 1.16, m	29.7	1.88, m 1.24, m	27.9	1.24, m 1.16, m
17	50.1	1.57, m	50.5	1.57, m	50.4	1.48, m	50.8	1.48, m
18	15.7	0.87, s	15.4	0.85, s	15.3	0.81, s	15.4	0.86, s
19	28.0	0.92, s	28.0	0.91, s	27.1	1.01, s	28.0	0.92, s
20	40.1	2.16, m	36.3	1.45, m	36.2	1.50, m	35.8	1.45, m
21	20.4	1.00, d (6.6)	18.9	0.91, d (6.6)	18.6	0.88, d (5.9)	18.7	0.91, d (5.3)
22	141.3	5.59, overlap	33.6	1.75, m 0.99, m	39.1	2.14, m 1.73, m	29.5	1.05, m 0.92, m
23	125.7	5.42, m	28.6	1.70, m 1.14, m	125.3	5.59, overlap	32.2	1.64, m 1.43, m
24	79.7	3.83, d (7.1)	79.6	3.27, d (9.8)	139.5	5.59, overlap	63.6	3.62, t (6.2)
25	72.9	–	73.2	–	70.7	–	17.8	0.81, s
26	26.3	1.20, s	26.5	1.20, s	29.9	1.31, s	27.2	1.03, s
27	23.7	1.14, s	23.2	1.15, s	30.0	1.31, s	25.5	1.14, s
28	17.8	0.81, s	17.8	0.80, s	20.5	0.85, s		
29	27.2	1.02, s	27.2	1.02, s	24.8	1.12, s		
30	25.4	1.13, s	25.4	1.13, s	19.9	0.88, s

Compound **2** was obtained as white solid and assigned a molecular formula of C_30_H_52_O_3_ (HRESIMS *m/z* 495.3622 [M + Cl]^−^, calcd for 495.3610), with two hydrogen atoms more than that of **1** (493.3447 [M + Cl]^−^). The ^1^H and ^13^C NMR spectra (Table 1) of **2** were very similar to **1**, except that two olefinic protons of **1** were replaced by two methylenes in **2**. Therefore, the structure of **2** was determined to be a hydrogenated derivative of **1** at C-22/C-23 double bond. The assignment was confirmed by the ^1^H–^1^H COSY correlations of CH_3_-21/H-20/CH_2_-22/CH_2_-23/H-24 and key HMBC correlations of the oxygenated methine proton at *δ*_H_ 3.31 (H-24) with C-22 and C-23, and of CH_3_-26 and CH_3_-27 with C-24 (*δ*_C_ 79.6). Similarly, the absolute configuration of C-24 in **2** was confirmed using the Snatzke’s method [9, 10]. The positive Cotton effect observed at 310 nm (Fig. 4) permitted the assignment of a 24*S* configuration for **2**. Thus, the structure of compound **2** was identified as cucurbita-5-ene-3*β*,24*S*,25-triol.

Compound **3** was isolated as white solid. Its molecular formula (C_30_H_50_O_3_), ascertained via high resolution ESI–MS analysis, indicated six degrees of unsaturation. Detailed analysis of the NMR (Table 1) and MS spectra led to the conclusion that the only difference between **3** and known compound **9** was that there is an epoxide group between C-5 (*δ*_C_ 66.8, s) and C-6 (*δ*_C_ 53.2, d) in **3** instead of a double bond between C-5 (*δ*_C_ 141.2, s) and C-6 (*δ*_C_ 121.4, d) in **9**. The epoxide group was elucidated by HMBC correlations of H-1, H-3, H-7, CH_3_-29 and CH_3_-30 with C-5, and of H-8 and H-10 with C-6, as well as the ^1^H–^1^H COSY correlations of H-6/H-7. The NOESY correlations of H-6/CH_3_-29 indicated the epoxy ring of **3** was in *β*-orientation. Thus, the structure of compound **3** was identified as cucurbita-5*β*,6*β*-epoxy-23-ene-3*β*, 25-diol.

Compound **4** was obtained as white solid and assigned a molecular formula of C_27_H_46_O_2_, (HRESIMS *m/z* 403.3594 [M + H]^+^, calcd for 403.3571), indicating five degrees of unsaturation. In the ^1^H NMR spectrum (Table 1), the signals of five tertiary methyl groups (*δ*_H_ 1.14, 1.03, 0.92, 0.86, 0.81) and one secondary methyl group (*δ*_H_ 0.91, 3H, d, *J* = 5.3 Hz) were observed. The ^13^C NMR spectrum of **4** showed signals for 27 carbons due to six methyl groups, two olefinic carbons, ten methylenes (including an oxygenated one), five methines (including an oxygenated one), and four quaternary carbons. Detailed comparison of the ^13^C NMR spectrum of **4** with that of **2** displayed similarities in rings A–D, except for the absence of the signals for C-25, 26, 27. These evidences revealed that compound **4** is a rare 25,26,27-trinorcucurbitane triterpenoid. This can be confirmed via the ^1^H–^1^H COSY correlations of H_3_-21/H-20/H_2_-22/H_2_-23/H_2_-24. Thus, the structure of compound **4** was identified as 25,26,27-trinorcucurbita-5-ene-3*β*,24-diol.

Compound **5** was isolated as white solid. Its molecular formula (C_27_H_44_O_3_), ascertained via high resolution ESI–MS analysis, indicated six degrees of unsaturation. Analysis of the ^1^H and ^13^C NMR spectroscopic data of **5** (Table 2) indicated a structural similarity with compound **4**, except that compound **5** has a carboxyl (*δ*_C_ 178.8, C-24) instead of hydroxyl methyl signals in **4**. The deduction was confirmed via the HMBC correlations from H-22, H-23 to the carboxyl carbon (C-24). The relative configurations of **5** were evidenced to be identical to those of **4** by analysis of NOESY spectrum. Thus, the structure of compound **5** was identified as 25,26,27-trinorcucurbita-5-ene-3*β*-ol-24-acid (Table 3).

**Table 2:** ^1^H (500 MHz) and ^13^C (125 MHz) NMR spectroscopic data of compounds **5**-**8** in CDCl_3_

No.	Compound **5**		Compound **6**		Compound **7**		Compound **8**	
	*δ*_C_	*δ*_H_ (*J* in Hz)	*δ*_C_	*δ*_H_ (*J* in Hz)	*δ*_C_	*δ*_H_ (*J* in Hz)	*δ*_C_	*δ*_H_ (*J* in Hz)
1	21.1	1.57, m 1.46, m	21.1	1.58, m 1.47, m	22.7	1.95, m 1.72, m	22.7	1.95, m 1.72, m
2	28.9	1.69, m 1.46, m	28.9	1.69, m 1.46, m	41.5	2.40, m 2.33, m	41.5	2.42, m 2.33, m
3	76.6	3.47, brt (2.5)	76.6	3.47, brt (2.5)	213.2	–	213.1	–
4	41.4	–	41.4	–	58.2	2.26, m	58.2	2.26, m
5	141.3	–	141.2	–	42.4	–	42.4	–
6	121.5	5.59, d (5.6)	121.5	5.59, d (5.7)	40.8	1.74, m 1.34, m	40.8	1.74, m 1.34, m
7	24.4	2.39, m 1.79, m	24.4	2.39, m 1.79, m	20.3	1.53, m 1.27, m	20.3	1.53, m 1.27, m
8	43.6	1.76, m	43.6	1.76, m	49.7	1.55, m	49.7	1.55, m
9	34.5	–	34.5	–	37.8	–	37.8	–
10	37.8	2.26, m	37.8	2.26, d (12.1)	59.0	1.58, m	59.0	1.58, m
11	32.3	1.64, m	32.3	1.66, m	36.6	1.43, m	36.7	1.44, m
		1.43, m		1.44, m		1.39, m		1.39, m
12	30.4	1.66, m 1.48, m	30.4	1.69, m 1.49, m	30.0	1.71, m 1.54, m	30.0	1.71, m 1.54, m
13	46.3	–	46.3	–	46.2	–	46.2	–
14	49.2	–	49.2	–	48.1	–	48.1	–
15	34.7	1.20, m 1.14, m	34.7	1.46, m 1.20, m	34.0	2.23, m 1.20, m	34.0	2.33, m 1.30, m
16	27.8	1.24, m 1.16, m	28.1	1.87, m 1.15, m	27.9	1.89, m 1.25, m	27.9	1.89, m 1.24, m
17	50.3	1.48, m	50.8	1.51, m	50.1	1.47, m	50.4	1.46, m
18	15.4	0.86, s	15.3	0.86, s	36.2	1.53, m	35.8	1.50, m
19	28.0	0.92, s	28.0	0.92, s	39.1	2.16, m; 1.75, m	31.9	1.44, m 0.95, m
20	35.5	1.48, m	33.1	1.57, m	125.4	5.59, overlap	31.5	1.63, m
								1.48, m
21	18.3	0.91, d (5.3)	18.9	0.93, d (5.3)	139.4	5.59, overlap	76.7	4.02, t (6.4)
22	30.9	2.39, m 2.26, m	39.4	1.72, m 1.23, m	70.7	–	147.4	–
23	31.1	1.81, m 1.30, m	61.0	3.68, m (2H)	6.8	0.87, d (6.5)	6.8	0.86, d(6.5)
24	178.8	–	17.8	0.81, s	14.6	0.72, s	14.6	0.72, s
25	17.8	0.81, s	27.2	1.02, s	18.5	0.85, s	18.5	0.85, s
26	27.2	1.03, s	25.5	1.14, s	19.2	0.78, s	19.2	0.78, s
27	25.4	1.14, s			15.8	0.88, s	15.8	0.87, s
28					18.6	0.89, d (6.5)	18.7	0.91, d(5.8)
29					30.0	1.30, s	17.2	1.72, s
30					29.9	1.31, s	111.4	4.93, m
								4.84, m

**Table 3:** Inhibitory effects of Compounds **1**-**9** against COX-2 in Vitro

Compounds	COX-2	Compounds	COX-2
	IC_50_ (μM)		IC_50_ (μM)
**1**	> 100	**6**	31.02 ± 2.64
**2**	18.94 ± 1.65	**7**	> 100
**3**	> 100	**8**	> 100
**4**	3.98 ± 0.32	**9**	> 100
**5**	19.48 ± 1.87	NS-398	4.14 ± 0.28

Analysis of HRESIMS spectrum ascribed compound **6** to a molecular formula C_26_H_44_O_2_ due to an adducting ion peak at *m/z* 389.3442 [M + H]^+^. The NMR data (Table 2) of **6** exhibited one methylene less than those of **4**, which can be confirmed by key ^1^H–^1^H COSY correlations of H-21/H-20/H-22/H-23 as well as HMBC correlation from hydroxyl methyl proton at *δ*_H_ 3.68 (2H, m) to C-20 (*δ*_C_ 33.1). Thus, the structure of compound **6** was identified as a rare 24,25,26,27-tetranorcucurbitane triterpenoid, and named 24,25,26,27-tetranorcucurbita-5-ene-3*β*,23-diol.

The molecular formula of **7**, C_30_H_50_O_2_, was determined due to HRESIMS adducting ion peak at *m/z* 443.3904 [M + H]^+^. The ^1^H NMR spectroscopic data (Table 2) gave two olefinic protons at *δ*_H_ 5.59 and eight methyls at *δ*_H_ 0.87 (d, 6.5 Hz), 0.72 (s), 0.85 (s), 0.78 (s), 0.88 (s), 0.89 (d, 6.5 Hz), 1.30 (s), 1.31 (s). The ^13^C NMR spectrum revealed the presence of 30 carbon resonances which were sorted into eight methyl carbons, nine methylenes, and seven methine carbons, and six quaternary carbons by DEPT NMR spectrum. Detailed comparison of the NMR data of **7** with those of maytefolin C [11] demonstrated that it possesses the same 18*R*-D:A-friedoeuphane skeleton, and differs from maytefolin C only at its side chain. The side chain of **7** was determined to be identical to that of known compound **9** by comparison of their ^1^H and ^13^C NMR chemical shifts (Table 2). This was further confirmed via the ^1^H–^1^H COSY correlations of H-18/H-28, H-18/H-19/H-20 and the key HMBC correlations from H-21, CH_3_-29, CH_3_-30 to C-22, and from H-20 to C-19 (Fig. 2). The relative configurations of **7** were assigned as shown in Fig. 3 by analysis of the NOESY spectrum (Fig. 3). Thus, the structure of compound **7** was identified as 18*R*-D:A-friedoeuph-20-ene-22-ol-3-one.

Compound **8** was obtained as yellow solid, and had the same molecular formula as **7** (C_30_H_50_O_2_), as ascertained via HRESIMS adducting ion peak at *m/z* 443.3924 [M + H]^+^. Detailed comparison of the NMR data with those of **7** revealed that **8** possessed a 18*R*-D:A-friedoeuphane skeleton as well, differing from **7** only in the positions of the double bond and the oxymethine at the side chain. The HMBC correlations from CH_3_-29 to two olefinic carbons at *δ*_C_ 147.4 and 111.4 disclosed that a terminal double bond was placed at C-22 and C-30 positions. A hydroxyl was substituted at C-21 due to key HMBC correlations of CH_3_-29 and H-30 with the oxygenated methine carbon at *δ*_C_ 76.7. The absolute configuration of C-21 was assigned as *S* on the basis of comparison of the chemical shifts of C-21 (*δ*_C_ 76.7) and H-21 (*δ*_H_ 4.02, 1H, t, *J *= 6.4 Hz) with those in literature [12]. Thus, the structure of compound **8** was identified as 18*R*-D:A-friedoeuph-22(30)-en-21*S*-ol-3-one.

All compounds were evaluated for their COX-2 inhibitory activities with NS-398 as a positive control. The results (Table 3) exhibited that compound **4** had the most potent inhibition against COX-2 with IC_50_ values of 3.98 ± 0.32 μM, while compounds **2**, **5** and **6** showed mild inhibitory effects with IC_50_ values of 18.94 ± 1.65, 19.48 ± 1.87 and 31.02 ± 2.64 μM. Compounds **1**–**6** and **9** share similar or even the same rings A, B, C, D, and the major difference is their side chains. Therefore, it seems that the side chain is the main factor to influence the inhibitions of compounds **1**–**6** and **9** against COX-2.

## Conclusion

In conclusion, this research led to the isolation of eight new triterpenoids and one known triterpenoid from the *A. latifolia,* in which compounds **4**–**6** are rare trinorcucurbitane or tetranorcucurbitane triterpenoids. It is the first report of cucurbitane-type triterpenoids from the genus *Ainsliaea*. Interestingly, compound **4** showed potent inhibition against COX-2 with IC_50_ values of 3.98 ± 0.32 μM. These results imply, except for sesquiterpenoids, triterpenoids may be another type of important chemical constituents being responsible for anti-inflammation in *Ainsliaea* species. Therefore, more attention should be paid to structural novel triterpenoids of *Ainsliaea* plants.

## Experimental Section

### General Experimental Procedures

Optical rotations were measured on a PerkineElmer 341 polarimeter. ^1^H and ^13^C NMR spectra were recorded on Bruker Avance-500 spectrometers. ESI–MS were measured on an Agilent LC/MSD Trap XCT spectrometer, and HRESIMS were performed on an Agilent 6520 Accurate-MS Q-TOF LC/MS system. A preparative column (ZORBAX-ODS GSA10250AP1301, C18, 5 μm, 250 × 10 mm) was used for semi-preparative HPLC (Shimadzu LC-2010A HT). TLC analysis was run on HSGF_254_ silica gel plates (10–40 μm, Yantai, China). Column chromatography (CC) was performed on silica (100–200, 200–300 mesh, Yantai, China), YMC-GEL ODS-A (50 μm, YMC, Japan), Sephadex LH-20 (Amersham Pharmacia Biotech AB, Uppsala, Sweden).

### Plant Material

The dried whole plants of *A. latifolia* were collected from Guiyang city of Guizhou province, PR China in September 2013, and authenticated by Prof. Long Qing-De, Department of Pharmacognosy, School of Pharmacy, Guiyang Medical University. An authentic specimen (No. 20130905) was deposited at the School of Pharmacy, Second Military Medical University.

### Extraction and Isolation

The dried whole plants of *A. latifolia* (15.0 kg) were powdered and extracted with EtOH-H_2_O (80:20, v/v) twice at room temperature, 48 h each time. The combined EtOH extracts were concentrated *in vacuo* to yield a crude extract (2.0 kg) which was then successfully partitioned with petroleum ether (PE), CHCl_3_, EtOAc, and MeOH, respectively, The CHCl_3_ fraction (105 g) was chromatographed on a silica gel column, eluting with gradient PE/EtOAc (100:1; 50:1; 20:1; 10:1; 5:1) to give six fractions (F1: 19.2 g, F2: 5.2 g, F3: 7.3 g, F4: 21.7 g, F5: 7.9 g, F6: 13.1 g). Fraction F2 was subjected to column chromatography (CC) over Sephadex LH-20 (MeOH) and silica gel to give compounds **7** (12.0 mg), **8** (4.2 mg). Fraction F3 was separated over Sephadex LH-20 (MeOH) followed by semi-preparative HPLC (CH_3_CN–H_2_O, 100:0), to yield **1** (3.0 mg), **2** (9.0 mg), and **3** (9.4 mg), respectively. Fraction F4 was subjected to ODS CC, eluted with a MeOH–H_2_O gradient, to yield 10 subfractions (F4A–F4 J). Subfraction F4B (507 mg) was subjected to CC over Sephadex LH-20 (MeOH) and silica gel to give compounds **4** (4.0 mg), **5** (4.2 mg), **6** (3.2 mg) and **9** (11.7 mg).

#### Cucurbita-5,22-diene-3β,24*S*,25-triol (**1**)

White solid; $\left[ \alpha \right]_{{\text{D}}}^{{20}}$ + 18.7 (*c* 0.10, CHCl_3_); UV (MeOH) λ_max_ (log ε) 204 (3.71) nm; For ^1^H NMR and ^13^C NMR spectroscopic data, see Table 1; HRESIMS *m/z* 493.3447 [M + Cl]^−^ (calcd for C_30_H_50_O_3_, 493.3454).

#### Cucurbita-5-ene-3β,24*S*,25-triol (**2**)

White solid; $\left[ \alpha \right]_{{\text{D}}}^{{20}}$ + 46.6 (*c* 0.30, CHCl_3_); UV (MeOH) λ_max_ (log ε) 204 (3.72) nm; For ^1^H NMR and ^13^C NMR spectroscopic data, see Table 1; HRESIMS *m/z* 495.3622 [M + Cl]^−^ (calcd for C_30_H_52_O_3_, 495.3610).

#### Cucurbita-5β,6β-epoxy-23-ene-3β,25-diol (**3**)

White solid; $\left[ \alpha \right]_{{\text{D}}}^{{20}}$ + 1.7 (*c* 0.13, CHCl_3_); UV (MeOH) λ_max_ (log ε) 201 (3.62), 203 (3.69), 231 (3.52) nm; For ^1^H NMR and ^13^C NMR spectroscopic data, see Table 1; HRESIMS *m/z* 493.3457 [M + Cl]^−^ (calcd for C_30_H_50_O_3_, 493.3454).

#### Cucurbita-5-ene-3β,24-diol (**4**)

White solid; $\left[ \alpha \right]_{{\text{D}}}^{{20}}$ + 48.0 (*c* 0.31, CHCl_3_); UV (MeOH) λ_max_ (log ε) 205 (3.73), 207 (3.71) nm; For ^1^H NMR and ^13^C NMR spectroscopic data, see Table 1; HRESIMS *m/z* 403.3594 [M + H]^+^ (calcd for C_27_H_46_O_2_, 403.3571).

#### Cucurbita-5-ene-3β-ol-24-acid (**5**)

White solid; $\left[ \alpha \right]_{{\text{D}}}^{{20}}$ + 32.7 (*c* 0.08, CHCl_3_); UV (MeOH) λ_max_ (log ε) 203 (3.64) nm; For ^1^H NMR and ^13^C NMR spectroscopic data, see Table 2; HRESIMS *m/z* 451.2980 [M + Cl]^−^ (calcd for C_27_H_44_O_3_, 451.2984).

#### Cucurbita-5-ene-3β,23-diol (**6**)

White solid; $\left[ \alpha \right]_{{\text{D}}}^{{20}}$ + 9.3 (*c* 0.11, CHCl_3_); UV (MeOH) λ_max_ (log ε) 205 (3.54) nm; For ^1^H NMR and ^13^C NMR spectroscopic data, see Table 2; HRESIMS *m/z* 389.3442 [M + H]^+^ (calcd for C_26_H_44_O_2_, 389.3414).

#### 18R-D:A-friedoeuph-20-ene-22-ol-3-one (**7**)

White solid; $\left[ \alpha \right]_{{\text{D}}}^{{20}}$ – 17.4 (*c* 0.37, CHCl_3_); UV (MeOH) λ_max_ (log ε) 207 (3.18), 231 (3.28) nm; For ^1^H NMR and ^13^C NMR spectroscopic data, see Table 2; HRESIMS *m/z* 443.3904 [M + H]^+^ (calcd for C_30_H_50_O_2_, 443.3884).

#### 18R-D:A-friedoeuph-22-en-21*S*-ol-3-one (**8**)

White solid; $\left[ \alpha \right]_{{\text{D}}}^{{20}}$ – 37.9 (*c* 0.15, CHCl_3_); UV (MeOH) λ_max_ (log ε) 201 (3.44), 203 (3.54) nm; For ^1^H NMR and ^13^C NMR spectroscopic data, see Table 2; HRESIMS *m/z* 443.3924 [M + H]^+^ (calcd for C_30_H_50_O_2_, 443.3884).

#### Cucurbita-5,23-diene-3*β*,25-diol (**9**)

White solid, C_30_H_50_O_2_; ^1^H NMR (500 MHz, CDCl_3_): *δ*_H_ 0.79 (3H, CH_3_-30), 0.85 (3H, s, CH_3_-18), 0.87 (3H, d, *J* = 5.8 Hz, CH_3_-21), 0.91 (3H, s, CH_3_-19), 1.02 (3H, s, CH_3_-28), 1.13 (3H, s, CH_3_-29), 1.30 (2 × CH_3_, s, CH_3_-26, 27), 2.26 (1H, d, *J* = 12.1 Hz, H-10), 2.38 (1H, m, H-7), 3.47 (1H, br.t, *J* = 2.5 Hz, H-3), 5.58 (3H, m, H-6, 23, 24); ^13^C NMR (125 MHz, CDCl_3_): *δ*_C_ 21.1 (t, C-1), 28.9 (t, C-2), 76.6 (d, C-3), 41.4 (s, C-4), 141.2 (s, C-5), 121.4 (d, C-6), 24.3 (t, C-7), 43.6 (d, C-8), 34.5 (s, C-9), 37.8 (d, C-10), 32.3 (t, C-11), 30.3 (t, C-12), 46.3 (s, C-13), 49.2 (s, C-14), 34.8 (t, C-15), 27.8 (t, C-16), 50.1 (d, C-17), 15.4 (q, C-18), 28.0 (q, C-19), 36.2 (d, C-20), 18.7 (q, C-21), 39.1 (t, C-22), 125.5 (d, C-23), 139.4 (d, C-24), 70.7 (s, C-25), 29.8 (q, C-26), 29.9 (q, C-27), 17.8 (q, C-30), 27.2 (q, C-28), 25.4 (q, C-29); ESI–MS: *m/z* 465 [M + Na]^+^ (positive), 441 [M − H]^−^ (negative).

### Determination of the Absolute Configuration of C-24 in Compounds **1** and **2**

According to the published literature [9, 10], a mixture of compound **1** (1.1 mg) and Mo_2_(OAc)_4_ (1.2 mg) was prepared for CD measurement. The mixture was kept for 30 min to form a stable chiral metal complex, the CD spectrum of which was then recorded. The observed sign of the diagnostic ICD (induced CD spectrum) curve at around 315 nm was correlated with the absolute configuration of C-24 in compound **1**. Compound **2** was also dealt with the same method as **1**.

### COX-2 Inhibitory Effect Assay

Cayman’s Colorimetric COX Inhibitor Screening Assay provides a convenient method for human recombinant COX-2 to screen isozyme-specific inhibitors. The assay measures the peroxidase component of COXs. The peroxidase activity is assayed colorimetrically by monitoring the appearance of oxidized N′,N,N,N′-tetramethyl-*p*-phenylenediamine (TMPD) at 590 nm. The COX-2 assay consisted of a 200 µL reaction mixture containing 150 µL assay buffer, 10 µL Heme, 10 µL COX-2, 20 µL Colorimetric Substrate, and 10 µL test solution (1, 5, 10, 20, 80, 100 µmol·L^−1^). The reactions were initiated by quickly adding 10 µL Arachidonic Acid, then incubating for 2 min at room temperature [13].

## Electronic supplementary material

Below is the link to the electronic supplementary material.

Supplementary material 1 (DOC 6352 kb)
