# Supplementary material for: Triterpenoids from Ainsliaea latifolia and Their Cyclooxyenase-2 (COX-2) Inhibitory Activities
Source: Nat Prod Bioprospect. 2019 Nov 30;10(1):13–21. doi: 10.1007/s13659-019-00228-x (PMC7046841; doi:10.1007/s13659-019-00228-x)
Supplement: Supplementary file 1 — Supplementary material 1 (DOC 6352 kb) [file 13659_2019_228_MOESM1_ESM.doc]

**Supporting Information**

**Triterpenoids from *Ainsliaea latifolia* and their cyclooxyenase-2 (COX-2) inhibitory activities**

**Wen-Lin Yuan^1,±^ · Xue-Yun Dong^1,4,±^ · Zheng-Rui Huang^3^ · Si-Jia Xiao^1^ ·Ji Ye^1^ · Xin-Hui Tian^2^ · Hui-Liang Li^1^ · Yun-Heng Shen^1^,*****. Wei-Dong Zhang ^1, 2^,[[1]](#footnote-2)***

***Dedicated to Professor Han-Dong Sun on the occasion of his 80th birthday***

^1^ Department of Phytochemistry, School of Pharmacy, Naval Medical University (Second Military Medical University), Shanghai 200433, China

^2^ Interdisciplinary Science Research Institute, Shanghai University of Traditional Chinese Medicine, Shanghai 201203, China

^3^ Department of Applied Chemistry, Xi’an University of Technology, Xi’an 710048, China

^4^ School of Pharmacy, Fujian University of Traditional Chinese Medicine, Fujian 350108, China

**Electronic supplementary material** The online version of this article (<https://doi.org/>) contains supplementary, which is available to authorized users.

| **No.** | **Content** | **Page** |
| --- | --- | --- |
| 1 | Figure S1. ^1^H NMR spectrum of compound **1** | 1 |
| 2 | Figure S2. ^13^C NMR spectrum of compound **1** | 1 |
| 3 | Figure S3. DEPT spectrum of compound **1** | 2 |
| 4 | Figure S4. ^1^H-^1^H COSY spectrum of compound **1** | 2 |
| 5 | Figure S5. HSQC spectrum of compound **1** | 3 |
| 6 | Figure S6. HMBC spectrum of compound **1** | 3 |
| 7 | Figure S7. NOESY spectrum of compound **1** | 4 |
| 8 | Figure S8. ^1^H NMR spectrum of compound **2** | 4 |
| 9 | Figure S9. ^13^C NMR spectrum of compound **2** | 5 |
| 10 | Figure S10. DEPT spectrum of compound **2** | 5 |
| 11 | Figure S11. ^1^H-^1^H COSY spectrum of compound **2** | 6 |
| 12 | Figure S12. HSQC spectrum of compound **2** | 6 |
| 13 | Figure S13. HMBC spectrum of compound **2** | 7 |
| 14 | Figure S14. NOESY spectrum of compound **2** | 7 |
| 15 | Figure S15. ^1^H NMR spectrum of compound **3** | 8 |
| 16 | Figure S16. ^13^C NMR spectrum of compound **3** | 8 |
| 17 | Figure S17. DEPT spectrum of compound **3** | 9 |
| 18 | Figure S18. ^1^H-^1^H COSY spectrum of compound **3** | 9 |
| 19 | Figure S19. HSQC spectrum of compound **3** | 10 |
| 20 | Figure S20. HMBC spectrum of compound **3** | 10 |
| 21 | Figure S21. NOESY spectrum of compound **3** | 11 |
| 22 | Figure S22. ^1^H NMR spectrum of compound **4** | 11 |
| 23 | Figure S23. ^13^C NMR spectrum of compound **4** | 12 |
| 24 | Figure S24. DEPT spectrum of compound **4** | 12 |
| 25 | Figure S25. ^1^H-^1^H COSY spectrum of compound **4** | 13 |
| 26 | Figure S26. HSQC spectrum of compound **4** | 13 |
| 27 | Figure S27. HMBC spectrum of compound **4** | 14 |
| 28 | Figure S28. NOESY spectrum of compound **4** | 14 |
| 29 | Figure S29. ^1^H NMR spectrum of compound **5** | 15 |
| 30 | Figure S30. ^13^C NMR spectrum of compound **5** | 15 |
| 31 | Figure S31. DEPT spectrum of compound **5** | 16 |
| 32 | Figure S32. ^1^H-^1^H COSY spectrum of compound **5** | 16 |
| 33 | Figure S33. HSQC spectrum of compound **5** | 17 |
| 34 | Figure S34. HMBC spectrum of compound **5** | 17 |
| 35 | Figure S35. NOESY spectrum of compound **5** | 18 |
| 36 | Figure S36. ^1^H NMR spectrum of compound **6** | 18 |
| 37 | Figure S37. ^13^C NMR spectrum of compound **6** | 19 |
| 38 | Figure S38. DEPT spectrum of compound **6** | 19 |
| 39 | Figure S39. ^1^H-^1^H COSY spectrum of compound **6** | 20 |
| 40 | Figure S40. HSQC spectrum of compound **6** | 20 |
| 41 | Figure S41. HMBC spectrum of compound **6** | 21 |
| 42 | Figure S42. NOESY spectrum of compound **6** | 21 |
| 43 | Figure S43. ^1^H NMR spectrum of compound **7** | 22 |
| 44 | Figure S44. ^13^C NMR spectrum of compound **7** | 22 |
| 45 | Figure S45. DEPT spectrum of compound **7** | 23 |
| 46 | Figure S46. ^1^H-^1^H COSY spectrum of compound **7** | 23 |
| 47 | Figure S47. HSQC spectrum of compound **7** | 24 |
| 48 | Figure S48. HMBC spectrum of compound **7** | 24 |
| 49 | Figure S49. NOESY spectrum of compound **7** | 25 |
| 50 | Figure S50. ^1^H NMR spectrum of compound **8** | 25 |
| 51 | Figure S51. ^13^C NMR spectrum of compound **8** | 26 |
| 52 | Figure S52. DEPT spectrum of compound **8** | 26 |
| 53 | Figure S53. ^1^H-^1^H COSY spectrum of compound **8** | 27 |
| 54 | Figure S54. HSQC spectrum of compound **8** | 27 |
| 55 | Figure S55. HMBC spectrum of compound **8** | 28 |
| 56 | Figure S56. NOESY spectrum of compound **8** | 28 |
| 57 | Figure S57. ^1^H NMR spectrum of compound **9** | 29 |
| 58 | Figure S58. ^13^C and DEPT NMR spectra of compound **9** | 29 |


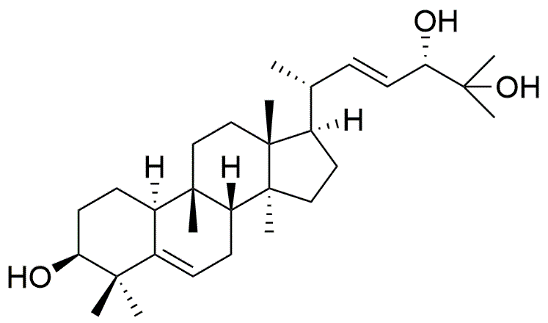


**Figure S1.** ^1^H NMR spectrum of compound **1**

**Figure S2.** ^13^C NMR spectrum of compound **1**

**Figure S3.** DEPT spectrum of compound **1**

**Figure S4.** ^1^H-^1^H COSY spectrum of compound **1**

**Figure S5.** HSQC spectrum of compound **1**

**Figure S6.** HMBC spectrum of compound **1**

**Figure S7.** NOESY spectrum of compound **1**


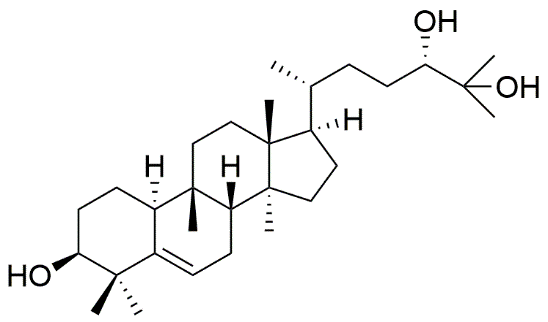


**Figure S8.** ^1^H NMR spectrum of compound **2**

**Figure S9.** ^13^C NMR spectrum of compound **2**

**Figure S10.** DEPT spectrum of compound **2**

**Figure S11.** ^1^H-^1^H COSY spectrum of compound **2**

**Figure S12.** HSQC spectrum of compound **2**

**Figure S13.** HMBC spectrum of compound **2**

**Figure S14.** NOESY spectrum of compound **2**


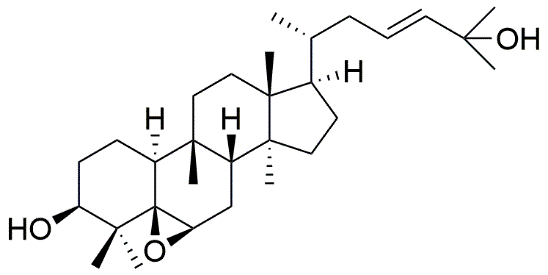


**Figure S15.** ^1^H NMR spectrum of compound **3**

**Figure S16.** ^13^C NMR spectrum of compound **3**

**Figure S17.** DEPT spectrum of compound **3**

**Figure S18.** ^1^H-^1^H COSY spectrum of compound **3**

**Figure S19.** HSQC spectrum of compound **3**

**Figure S20.** HMBC spectrum of compound **3**

**Figure S21.** NOESY spectrum of compound **3**


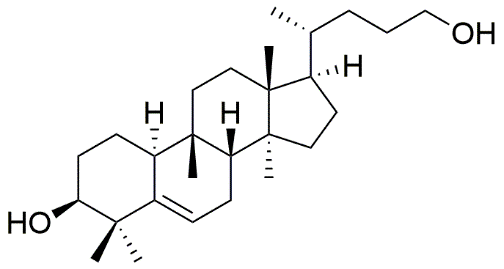


**Figure S22.** ^1^H NMR spectrum of compound **4**

**Figure S23.** ^13^C NMR spectrum of compound **4**

**Figure S24.** DEPT spectrum of compound **4**

**Figure S25.** ^1^H-^1^H COSY spectrum of compound **4**

**Figure S26.** HSQC spectrum of compound **4**

**Figure S27.** HMBC spectrum of compound **4**

**Figure S28.** NOESY spectrum of compound **4**


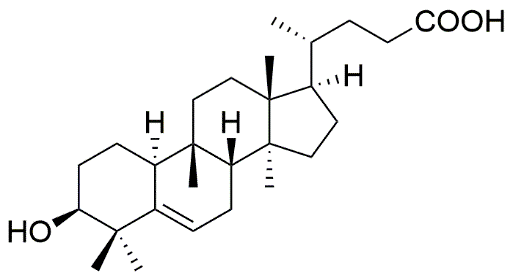


**Figure S29.** ^1^H NMR spectrum of compound **5**

**Figure S30.** ^13^C NMR spectrum of compound **5**

**Figure S31.** DEPT spectrum of compound **5**

**Figure S32.** ^1^H-^1^H COSY spectrum of compound **5**

**Figure S33.** HSQC spectrum of compound **5**

**Figure S34.** HMBC spectrum of compound **5**

**Figure S35.** NOESY spectrum of compound **5**


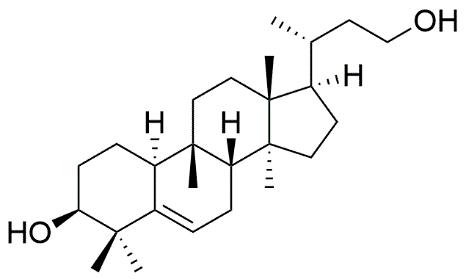


**Figure S36.** ^1^H NMR spectrum of compound **6**

**Figure S37.** ^13^C NMR spectrum of compound **6**

**Figure S38.** DEPT spectrum of compound **6**

**Figure S39.** ^1^H-^1^H COSY spectrum of compound **6**

**Figure S40.** HSQC spectrum of compound **6**

**Figure S41.** HMBC spectrum of compound **6**

**Figure S42.** NOESY spectrum of compound **6**


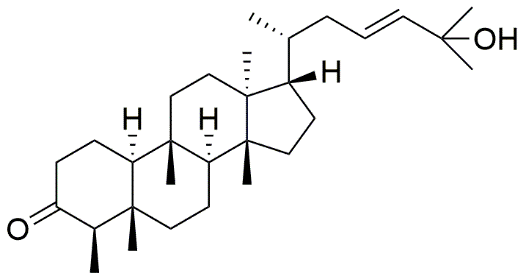


**Figure S43.** ^1^H NMR spectrum of compound **7**

**Figure S44.** ^13^C NMR spectrum of compound **7**

**Figure S45.** DEPT spectrum of compound **7**

**Figure S46.** ^1^H-^1^H COSY spectrum of compound **7**

**Figure S47.** HSQC spectrum of compound **7**

**Figure S48.** HMBC spectrum of compound **7**

**Figure S49.** NOESY spectrum of compound **7**


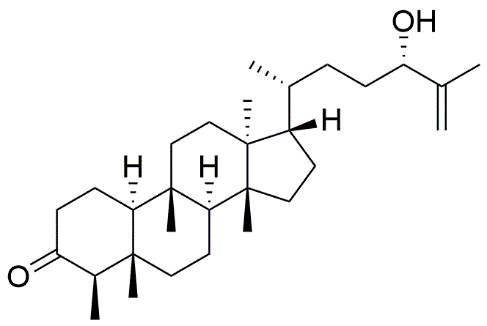


**Figure S50.** ^1^H NMR spectrum of compound **8**

**Figure S51.** ^13^C NMR spectrum of compound **8**

**Figure S52.** DEPT spectrum of compound **8**

**Figure S53.** ^1^H-^1^H COSY spectrum of compound **8**

**Figure S54.** HSQC spectrum of compound **8**

**Figure S55.** HMBC spectrum of compound **8**

**Figure S56.** NOESY spectrum of compound **8**

**
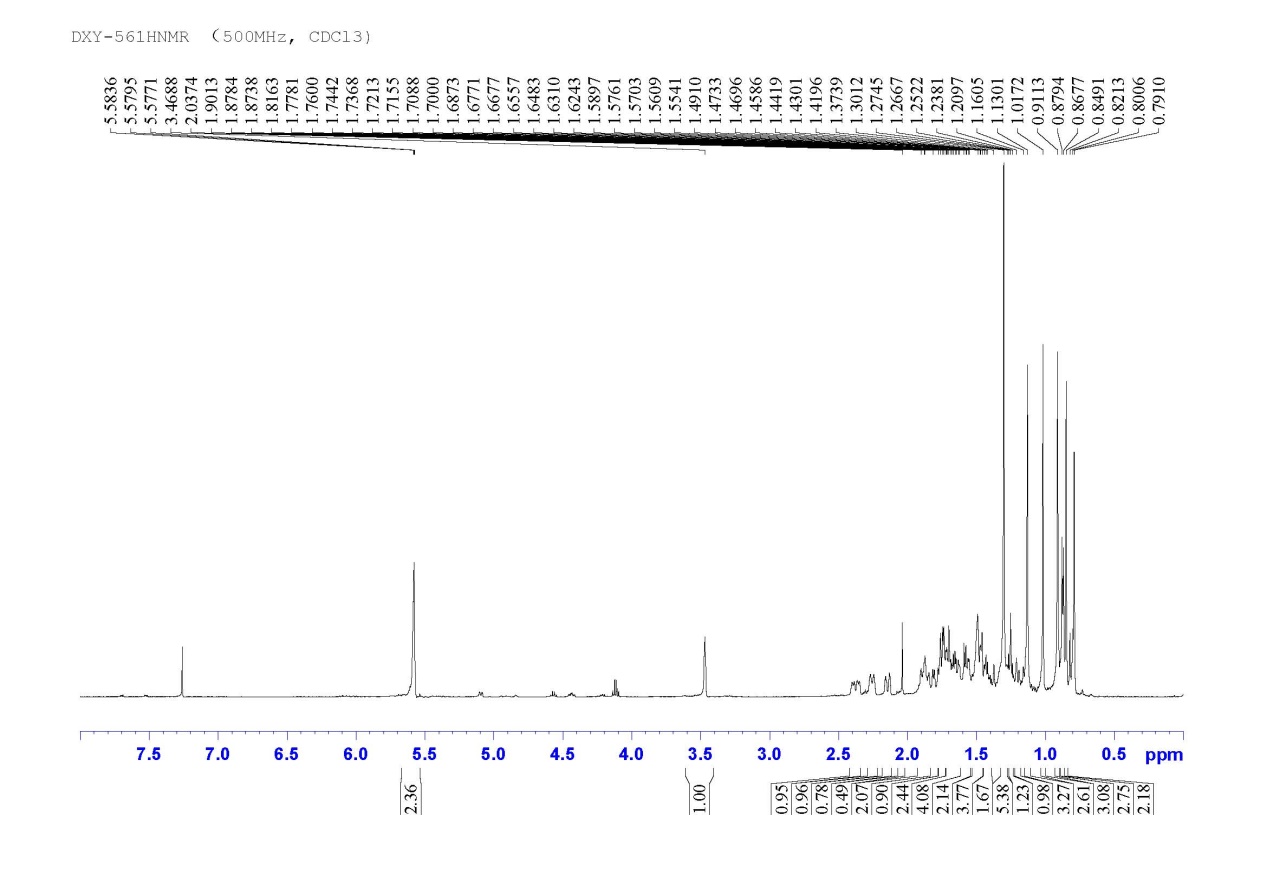
**

Figure S57. ^1^H NMR spectrum of compound **9**

**
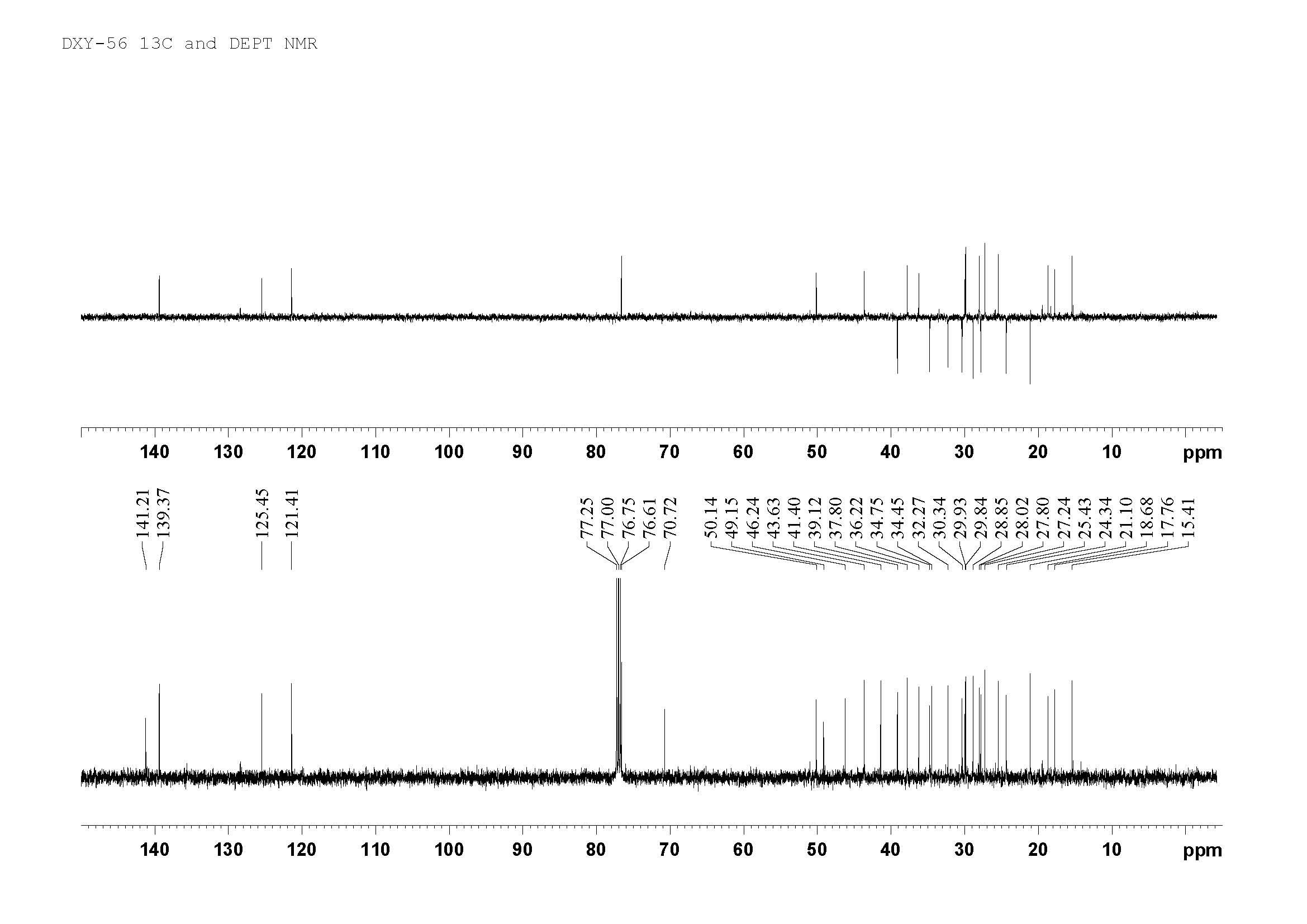
**

Figure S58. ^13^C and DEPT NMR spectra of compound **9**

1. ^*^ **Yun-Heng Shen (**[**shenyunheng@hotmail.com**](mailto:shenyunheng@hotmail.com)**)**

   **Wei-Dong Zhang(**[**wdzhangy@hotmail.com**](mailto:wdzhangy@hotmail.com)**)**

   **± These authors contributed equally to this work** [↑](#footnote-ref-2)
